# Low virulent infectious salmon anaemia virus (ISAV) replicates and initiates the immune response earlier than a highly virulent virus in Atlantic salmon gills

**DOI:** 10.1186/s13567-014-0083-x

**Published:** 2014-08-21

**Authors:** Alastair J A McBeath, Yee Mai Ho, Maria Aamelfot, Malcolm Hall, Debes H Christiansen, Turhan Markussen, Knut Falk, Iveta Matejusova

**Affiliations:** Marine Scotland Science, Marine Laboratory, Aberdeen, Scotland UK; Norwegian Veterinary Institute, Oslo, Norway; Food and Veterinary Authority, Tórshavn, Faroe Islands

## Abstract

Observations from the field and experimental evidence suggest that different strains of infectious salmon anaemia virus (ISAV) can induce disease of varying severity in Atlantic salmon. Variation in host mortality and dissemination of ISAV isolates with high and low virulence was investigated using immersion challenge; from which mortality, pathological, immunohistochemical and preliminary molecular results have been previously published. Here, real-time RT-PCR analysis and statistical modelling have been used to further investigate variation in virus load and the response of four select immune genes. Expression of type I and II interferon (IFN), Mx and γIFN induced protein (γIP) to high and low pathogenic virus infection were examined in gill, heart and anterior kidney. In addition, a novel RNA species-specific assay targeting individual RNA types was used to investigate the separate viral processes of transcription and replication. Unexpectedly, the low virulent ISAV (LVI) replicated and transcribed more rapidly in the gills compared to the highly virulent virus (HVI). Subsequently LVI was able to disseminate to the internal organs more quickly and induced a more rapid systemic immune response in the host that may have offered some protection. Contrary to this, HVI initially progressed more slowly in the gills resulting in a slower generalised infection. However HVI ultimately reached a higher viral load and induced a greater mortality.

**Electronic supplementary material:**

The online version of this article (doi:10.1186/s13567-014-0083-x) contains supplementary material, which is available to authorized users.

## Introduction

Infectious salmon anaemia virus (ISAV) is an aquatic orthomyxovirus that infects Atlantic salmon. The virus can cause serious disease problems resulting in substantial financial implications for the aquaculture industry. Infectious salmon anaemia (ISA) has occurred annually in Norway [1] since its discovery in 1984 [2] and has also been reported in North America [3-5], Scotland [6,7], Faroe Islands [8,9] and Chile [10]. ISAV is enveloped and possesses a genome of 8 negative-sense single-stranded RNA segments, coding for at least 10 proteins, similar to that of influenza virus [11,12]. Segments 1–4 produce the proteins that comprise the viral ribonucleoprotein core. Segments 5 and 6 encode the fusion (F) [13] and haemagglutinin-esterase (HE) [14,15] surface glycoproteins respectively. Segment 7 possesses two open reading frames (ORFs), the first of which is thought to produce a minor or non-structural protein (NS) [16] that interferes with interferon (IFN) and IFN induced systems [17,18]. The second ORF, generated via splicing, is believed to encode the nuclear export protein (NEP) [19]. Segment 8 also contains two ORFs; ORF1 encodes the major structural matrix (M) protein [14,16] and ORF2 is believed to produce a minor structural protein also with IFN antagonising activity [17]. The M protein is the most abundant protein in the virion [20] and therefore RNA of this segment is readily targeted in diagnostic tests [21].

The pathogenicity of ISAV is a multifactorial trait dependent on the function of viral proteins, interactions with host immune responses as well as various environmental factors. Little is known regarding the specific interplay of these aspects and how they influence ISAV infection. Nevertheless there are multiple reports that different strains of ISAV display different levels of virulence and associated mortality [22-26]. A deletion within the highly polymorphic region (HPR) of segment 6 has been suggested to be an important virulence marker [25,27]. All pathogenic strains reported to date contain an HPR deletion with respect to the putatively ancestral, non-virulent HPR0 virus [9,28]. In addition, substitutions and insertions adjacent to the putative cleavage site in the F protein have also been linked to virulence [27,29]. However, even isolates with identical HPR and F protein sequences have shown variation in virulence [25,27] indicating additional factors are also important.

Vertebrate nucleated cells rapidly secrete the cytokine molecules interferon (IFN) in response to viral infection. In turn, IFNs activate complex signalling pathways inducing the expression of hundreds of direct and indirect antiviral genes [for review see [30,31]]. In fish, both the Type I and Type II IFN systems are now known to be imperative in the innate and adaptive antiviral responses respectively [31,32]. ISAV is a potent inducer of immune genes yet the response offers little protection [25,33,34]. Intraperitoneal injection of a highly virulent ISAV indicated significant monophasic induction of several immune genes in kidney on day 6 post-infection, followed by increased virus production and high mortality [33]. The antiviral protein Mx has become a direct indicator of the innate Type I IFN response, induced by IFNs α and β in a variety of cell types. Similarly, the IFNγ-induced protein (γIP) gene can be used as an indicator of the adaptive Type II IFN response, stimulated by γIFN specifically in immune cells. The expression of these interferon stimulated genes (ISGs) as induced products of each system showed both the innate and adaptive responses were stimulated concurrently by ISAV injection [33]. More recently, cell cultures infected with ISAV of high or low virulence have indicated variations in immune response between strains [35,36].

The present study utilises an immersion infection of Atlantic salmon with two ISAV strains previously classed as low (LVI) and high virulence (HVI) [23,37]. The mortality, pathology, immunohistochemistry (IHC) and initial virus segment 8 qPCR results observed following the challenge are presented in McBeath et al. [37]. Here, the molecular results are analysed in more detail using statistical modelling of the qPCR data of viral segments 7 and 8 obtained from gill, heart and anterior kidney samples. The separate viral processes of replication and transcription were also investigated individually for both viruses using novel RNA species-specific assays based on a recently published method [38]. In addition, the expression levels of four host immune markers were also monitored and statistically analysed to determine if infection with LVI or HVI had variable effects on the immune system. Ultimately, LVI was shown to replicate more rapidly in the gills resulting in more rapid dissemination and immune response throughout the host, yet HVI reached higher viral loads, causing a more serious infection and inducing greater mortality. The results of this study contribute to our understanding of the pathogenesis of this important pathogen in Atlantic salmon aquaculture.

## Materials and methods

### Virus propagation, immersion challenge and organ sampling

Details regarding the virus production, infection challenge and organ sampling procedure have been described previously [37]. Briefly, fish were challenged by immersion with either 10^4^ TCID_50_ of highly virulent ISAV Glesvær 2/90 (HVI) or low virulent Can/F679/99 (LVI), or mock-infected with virus-free cell culture medium as uninfected negative controls. After 2 h, fish were transferred to sampling tanks (*n* = 75) or to observation tanks where cumulative mortalities for each treatment were observed (*n* = 20). Four fish were sampled 6 h post infection (pi) and on days 1 to 8, 10, 12, 14, 19 and 23 pi from each challenge group. Organ samples from gill, heart, and anterior-kidney, were collected in 1 mL RNAlater (Qiagen) and stored at −80 °C.

### Viral and immune gene expression real-time RT-PCR

RNA was extracted from 5 mg organ samples (gill, heart, anterior kidney) using the QIAsymphony® RNA robotic system (Qiagen) according to the manufacturers’ protocol and eluted in 100 μL RNase-free dH_2_O. The RNA was reverse transcribed to cDNA using the TaqMan® Reverse Transcription Reagents kit (Life Technologies, UK) with oligo-d(T)_16_ as described previously [33] using a 20 μL total reaction volume. Real-time RT-PCR assays were performed as described in McBeath et al. [37]. Primer and probe sequences (Additional file 1) for the assays targeting viral gene (no differentiation of ORFs in either assay) segments 7 (seg7) and 8 (seg8) from both European and North-American genogroups, immune markers Type I (α1 and α2), Type II interferon (IFN), Mx, γIFN-induced protein (γIP) and endogenous control elongation factor 1α (ELF), have been described previously [21,33]. Absolute quantitation of transcripts was carried out. The cycle crossing point (Cp) values were converted into expression values normalised against the reference gene, ELF, using the statistical standard curve method to produce relative expression ratios [39].

### Statistical analysis

The analysis focussed on modelling the scale of virus loads and immune responses for each ISAV isolate throughout the experiment. Statistical analyses were performed within the R statistical environment version 2.15.2 [40].

All observations greater than zero were transformed to log_10,_ converting the analysis into an evaluation of the variation in the scale of detectable expression for defined genes associated with two ISAV strains over time. The distributions of the transformed observations for each gene-strain-time combination are also more satisfactory approximations of the normal distribution. A preliminary inspection of the transformed observations suggested that there were differences in the dispersion of the scaled values at different times, and weights comprising the reciprocal of the variance for each strain-time combination were therefore calculated. These weights were used for the modelling of the changes in the scale of detectable gene expression for each strain over time using a locally-weighted running-line smoother [41]. Predicted values with their 95% confidence intervals (CI) were plotted. Plots exhibiting minimal overlap of CI for the two strains indicate differences in the trajectory of detectable expression for that gene over time between the two strains. A more formal evaluation of differences between strains was carried out by estimating the reduction in the residual sum of squares of nested linear models [42] describing gene expression on the potential explanatory variables of strain and time; *p*-values of ≤ 0.05 were categorised as statistically significant.

The association between the difference in the level of expression of the immunological markers (e.g. Mx) of the two strains with the difference in the load of the two strains (evaluated using both the segment 7 and segment 8) was evaluated for each tissue using Spearman’s rank correlation coefficient (r_s_) [43]. The statistical significance of associations is indicated by “star value” (*0.01 < *p* ≤ 0.05, **0.001 < *p* ≤ 0.01, ***p ≤ 0.001).

### RNA species specific real-time RT-PCR

RNA species specific reverse transcription (RT) was performed as described previously [38] using tagged primers for mRNA and cRNA (Additional file 1). Reverse transcription was performed at 65 °C using the Thermoscript RT kit (Life Technologies) according to manufacturers’ protocols.

Real-time PCR was carried out using SensiFAST Sybr no-rox Master Mix (Bioline) and primer pairs consisting of one primer specific to the tag portion of the RT-tagged primers and the other specific to the appropriate viral sequence (Additional file 1). Reactions consisted of 1 × SensiFAST Sybr no-rox Master Mix, 10 μM each primer, 1 μL cDNA template in a 20 μL volume and were subjected to a 95 °C for 2 min pre-incubation, 45 cycles of 95 °C for 5 s, 62 °C for 10 s, 72 °C for 10 s, followed by melting curve analysis (95 °C for 5 s, 65 °C for 1 min, slow heating to 97 °C and cooling at 40 °C for 30 s) using a Roche Lightcycler LC480. The cycle crossing point (Cp) values were cross checked with melt curve data and samples were deemed positive if the melt curve temperature was within ± 0.5 °C of the expected value. Samples with no Cp value or a Cp value correlated with an incorrect melt curve temperature (i.e. indication of non-specificity) were classed as negative. Samples with dual melt curves were classed as positive if one was at the correct temperature. Due to the complication of non-specificity, no attempt was made to quantify the different RNA types.

## Results

### Experimental infection and mortality

In fish infected with HVI, mortality began on day 13 pi and the cumulative mortality reached 100% by day 23 pi. In the LVI group, mortality began on day 17 pi and cumulative mortality reached 20% by day 23 pi. All dead fish tested positive in kidney for ISAV segment 8 by real-time PCR. No mortality was observed in the uninfected negative control fish. Further details of the above results including pathology and immunohistochemistry (IHC) are presented in McBeath et al. [37].

### Viral kinetics profile by detection of ISAV segment 8

**Figure 1:**
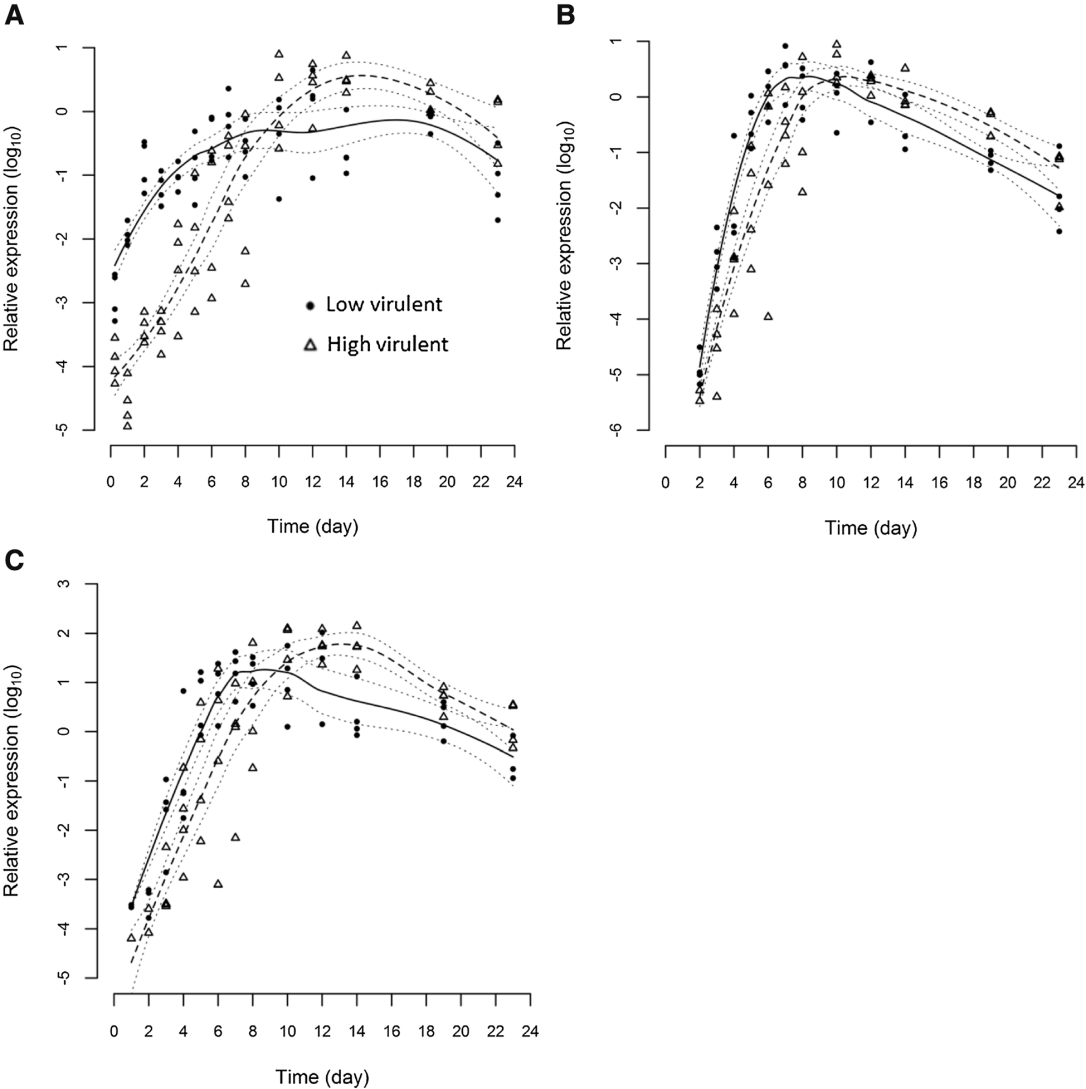
**Statistical modelling of ISAV segment 8.** Statistical modelling of viral segment 8 total RNA load profiles of high virulent (dashed line) and low virulent (solid line) strains measured by real-time RT-PCR from gill **(A)**, anterior kidney **(B)** and heart **(C)** tissues post-immersion infection at Day 0. Dotted lines indicate 95% confidence intervals.

All infected fish tested positive for segment 8 in the gills at every time point. In heart, no virus was detected at 6 h pi, 2/4 fish tested positive for LVI and 1/4 for HVI on day 1 pi. After 2 days, heart was positive in 3/4 fish infected with LVI and 2/4 infected with HVI. Kidney was also negative for segment 8 until day 2 pi when the LVI was detected in all 4 fish and HVI was detected in 2/4 fish. Thereafter from day 3 pi, both viruses were detected in all organ samples. Statistical modelling indicates that the time course of the 2 strains, as indicated by detection of segment 8, differ (*p* ≤ 0.05) for all 3 organs (Figure 1). This was most apparent in the early stages of the infection up to day 8 and especially notable in the gills (Figure 1A) during the first 4 days pi. Following day 8 pi, the LVI load almost stabilised in comparison to the HVI load which continued to increase further up to day 15 pi. All uninfected control fish were negative for ISAV.

### RNA species specific analysis of segment 8

The assays targeting RNA species corresponding specifically to the replication (cRNA) and transcription (mRNA) processes provided further evidence the LVI had successfully entered and replicated in the gills early in infection (Table 1). The results also suggest HVI has a slower uptake and/or entry into the gills and therefore progresses more slowly into generalised infection. In the LVI group, mRNA was detected in all fish (gills) at day 1 pi and thereafter until experimental end. In comparison, HVI mRNA was not first detected in gills until day 4 pi (in 3 fish) which correlated with the first large increase in HVI load by seg8 qPCR. Similarly, the LVI cRNA was also detected earlier in the gills than in the HVI group, although there was increased variation at the individual level in the latter. The difference in mRNA and cRNA production between strains was less pronounced in the kidney and the heart. In kidney, mRNA of both ISAV strains was detected at day 3 pi, however the number of positive individuals remained variable in the HVI group until day 7 pi. The first detection of cRNA in the kidney of the LVI group was in 1 fish on day 3 pi, followed by 3 fish on day 4 pi. The HVI cRNA was first detected in the kidney from 3 fish on day 4 pi (on days 5 and 6 also). In heart, the first mRNA detections for both viruses were from single fish on days 2 and 4 respectively, prior to all 4 fish on days 4 and 7 respectively. The first cRNA detections in heart occurred in 1 fish on day 3 pi in the LVI group and in 2 fish on day 4 in the HVI group.

**Table 1:** **RNA strand specific real-time RT-PCR**

	**Gill mRNA**		**Gill cRNA**		**Kidney mRNA**		**Kidney cRNA**		**Heart mRNA**		**Heart cRNA**	
**Day**	**LVI**	**HVI**	**LVI**	**HVI**	**LVI**	**HVI**	**LVI**	**HVI**	**LVI**	**HVI**	**LVI**	**HVI**
0.25	0	0	0	0	0	0	0	0	na	na	na	na
1	4	0	1	0	0	0	0	1	0	0	0	0
2	4	0	2	0	0	0	0	0	1	0	0	0
3	4	0	3	1	4	2	1	0	3	0	1	0
4	4	3	2	1	4	4	3	3	4	1	2	2
5	4	3	3	2	4	3	4	3	4	2	4	3
6	4	4	4	2	4	3	4	3	4	3	4	3
7	4	4	4	3	4	4	4	4	4	4	4	4
8	4	4	4	2	4	4	4	4	na	na	na	na
10-23	4	4	4	4	4	4	4	4	na	na	na	na

Number of fish (max. = 4) testing positive for mRNA or cRNA of low virulent (LVI) or highly virulent (HVI) virus using RNA strand specific real-time RT-PCR analysis of gill, anterior kidney and heart. na = not applicable.

As expected, the Cp values indicated cRNA was present in lower amounts than mRNA [38,44]. Cross-checking Cp results with melt curve data indicated non-specificity interfered with the mRNA assay when template levels were low (seg8 Cp > 32), as discussed previously [38]. Contrary to this, the cRNA assay was more specific, even at very low levels (seg8 Cp > 38) (data not shown).

### Immune gene expression

**Figure 2:**
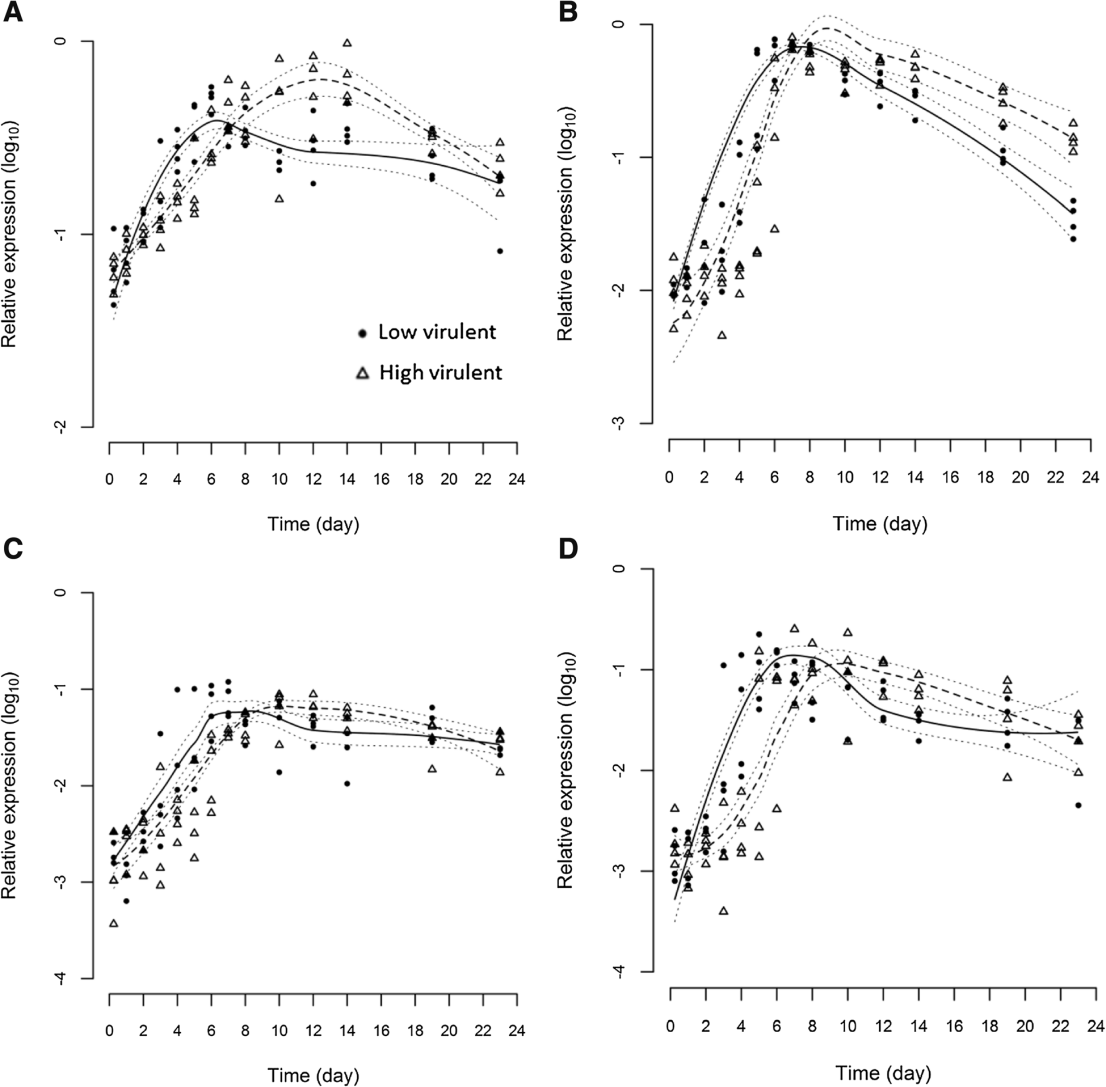
**Statistical modelling of immune genes in gill.** Statistical modelling of 4 immune marker expression profiles, Type I IFN **(A)**, Mx **(B)**, Type II IFN **(C)** and γIP **(D)** in gills of fish infected with high virulent (dashed line) or low virulent (solid line) strains measured by real-time RT-PCR. Dotted lines indicate 95% confidence intervals.

**Figure 3:**
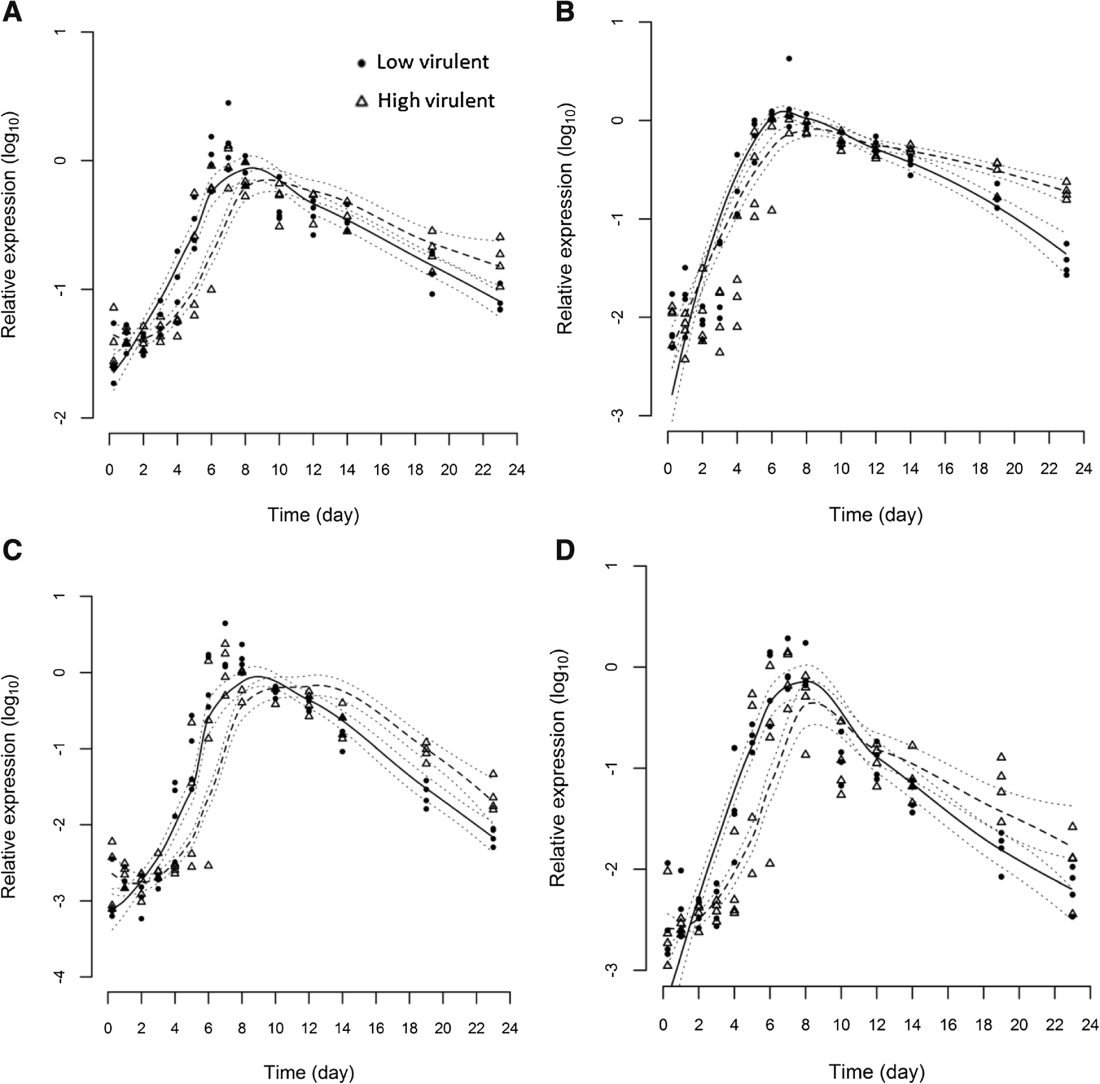
**Statistical modelling of immune genes in kidney.** Statistical modelling of 4 immune marker expression profiles, Type I IFN **(A)**, Mx **(B)**, Type II IFN **(C)** and γIP **(D)** in anterior kidney of fish infected with high virulent (dashed line) or low virulent (solid line) strains measured by real-time PCR. Dotted lines indicate 95% confidence intervals.

**Figure 4:**
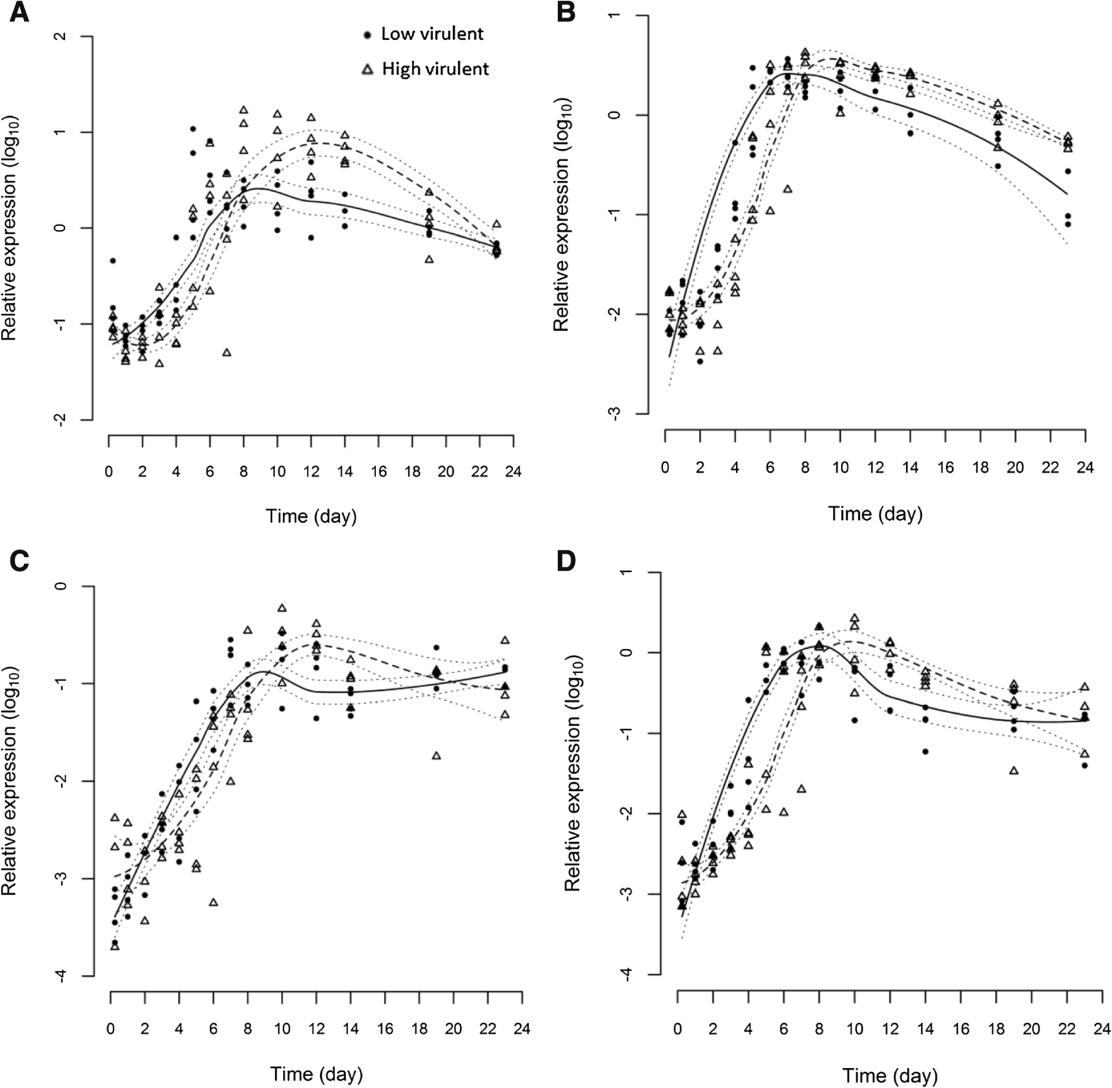
**Statistical modelling of immune genes in heart.** Statistical modelling of 4 immune marker expression profiles, Type I IFN **(A)**, Mx **(B)**, Type II IFN **(C)** and γIP **(D)** in heart of fish infected with high virulent (dashed line) or low virulent (solid line) strains measured by real-time RT-PCR. Dotted lines indicate 95% confidence intervals.

The expression of four immune markers, Type I and II IFN, Mx and γIP, was measured in gills, heart and anterior kidney to provide information on both innate and adaptive immune responses upon infection with the two different ISAV strains. All immune genes in infected fish were strongly up-regulated in comparison to the negative control fish throughout the experiment (Table 2). The maximum increase in transcript levels for all four immune gene markers to both ISAV strains was higher in kidney and heart compared to gills. The highest maximum increase was observed for Type II IFN in kidney. Statistical modelling indicates that the time course of expression for all four genes differ between the two strains (*p* ≤ 0.05) in gill (Figure 2), anterior kidney (Figure 3) and heart (Figure 4) indicating a differential immunological host response to the two viruses. In all cases, the immune genes were stimulated significantly more in the LVI group than the HVI group up to day 7 or 8 pi. In contrast, after day 8, the immune genes were expressed at higher levels in the HVI fish.

**Table 2:** **Fold increase in expression of immune genes**

	**Gill**			**Kidney**			**Heart**		
**Gene**	**LVI**	**HVI**	**Control**	**LVI**	**HVI**	**Control**	**LVI**	**HVI**	**Control**
Type I IFN	7.7	**10.4**	2.7	**49.6**	24.6	2.4	23.6	**100.5**	1.8
Mx	**71.6**	63	1.4	**213.4**	100	2.9	257.2	**271.9**	2.6
Type II IFN	30.2	**47**	5.9	**1443.2**	485	6.4	**394.5**	197.9	7.6
gIP	**93.2**	54	2.3	242.5	**255**	4.1	290.7	**424.3**	9.8

Fold increase from 6 h to the maximum peak of immune gene expression (on any given day) post-infection (pi) for fish infected with ISAV of either low (LVI) or high (HVI) virulence, or cell culture media to serve as negative controls. Figures in bold indicate the highest fold-induction for each gene in each organ.

### Segment 8 and immune gene correlation analysis

The associations between the difference in virus segment 8 of the low and the highly virulent viruses and the difference in immune gene expression response to each strain were investigated using correlation. The rationale for this is that a positive association between an increasing difference in load between the two strains and an increasing immune response is indicative of a possible causal relationship between viral load and immune response. Association of the differences between the segment 8 viral load and the expression of all immune genes in the gill and heart for both LVI and HVI (Table 3) were observed (*p* ≤ 0.05), indicating an association between viral load as measured by segment 8 RNA quantity and immune gene expression in these organs.

**Table 3:** **Correlation analysis**

	**Segment 8**			**Segment 7**		
**Immune gene**	**Gill**	**Kidney**	**Heart**	**Gill**	**Kidney**	**Heart**
**Mx**	0.75**	0.9***	0.9***	0.23	0.72**	0.12
**γIP**	0.61*	0.43	0.96***	−0.002	0.46	0.12
**Type I IFN**	0.61*	0.43	0.86***	−0.002	0.56*	−0.002
**Type II IFN**	0.82***	0.55*	0.78**	0.49	0.56*	0.2

Correlation coefficients (r_s_) for differences between strains with respect to virus burden and immune response. (*0.01 < *p* ≤ 0.05, **0.001 < *p* ≤ 0.01, ***p ≤ 0.001).

### Segment 7

**Figure 5:**
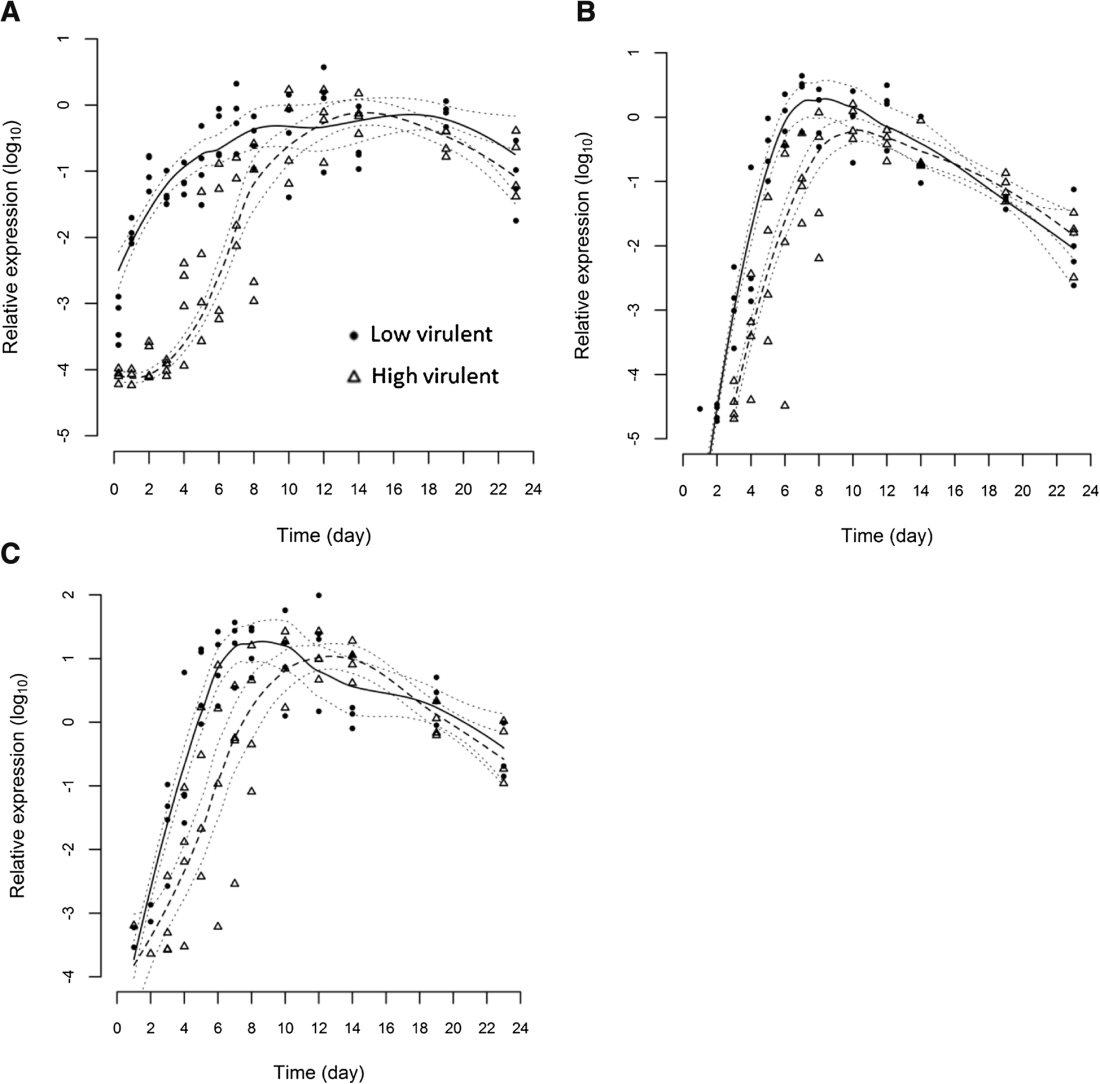
**Statistical modelling of ISAV segment 7.** Statistical modelling of viral segment 7 total RNA load profiles of high virulent (dashed line) and low virulent (solid line) strains measured by real-time RT-PCR from gill **(A)**, anterior kidney **(B)** and heart **(C)** tissues. Dotted lines indicate 95% confidence intervals.

Expression profiles for genomic segment 7 in gill, kidney and heart (Figure 5) were similar overall to those of segment 8, although a higher quantity of segment 7 was produced by LVI than HVI up to day 8 pi. This was more evident upon comparing a ratio of the two segments for each strain (Additional file 2). Following day 8 pi, unlike segment 8, there was little difference between the two viruses with regards to segment 7 RNA production as indicated by overlapping confidence intervals. In contrast to the results for segment 8, there was no evidence of an association in differences between the segment 7 viral loads and differences in immune response for gill and heart, although positive correlation (*p* ≤ 0.05) was observed in kidney (Table 3).

## Discussion

In the present study, organs from fish infected with low virulent ISAV (LVI) exhibited a higher viral load in the first 8 days of the infection compared to the highly virulent virus (HVI). This was most prominent in the gills during the first 4 days post-infection. The rapid increase in LVI segment 8 RNA load suggests the occurrence of a fast primary replication phase in the gill. The RNA species specific assays provided further support for this, indicating the LVI successfully gained fast entry to host gill cells, and was both actively replicating and transcribing in this early stage of infection. All four immune genes, representing both Type I and Type II IFN systems and two products thereof, were significantly differentially expressed when comparing the two ISAV isolates. This suggested differences in the host response towards the two virus infections. Statistical correlations provided further evidence suggesting a causal link between virus production (as measured by segment 8 RNA) and up-regulation of the studied immune genes in the gill and heart.

In an immersion challenge, all mucosal surfaces, including gill, skin, eye and gut, are possible entry points for microbial infections. The gills have previously been suggested as the main entry port for ISAV [45]. However fin and skin have both been implicated as important entry points for viral haemorrhagic septicaemia (VHSV), infectious haematopoietic necrosis virus (IHNV) and koi herpes virus (KHV) [46-48]. A cohabitation infection trial using the same HVI as the present study observed a relatively low level of infection in the gills, however few sampling points were used [34]. The currently presented viral load kinetics, backed up by the assays directly targeting cRNA and mRNA, suggest the presence of an early primary replication phase for the LVI in the gills, not observed for the HVI. Detection of both viruses in all fish at 6 h pi by seg8 real-time RT-PCR indicated rapid uptake of both viruses to the gills from the water. However, only the LVI amount displayed an early immediate increase which can initially be attributed to the rapid production of mRNA in the time points that follow. The replicative intermediate, cRNA, was also detected in some fish from day 1. As expected this was at much lower levels throughout the experiment [38,44]. Therefore the production of cRNA had a negligible effect on the overall viral load compared to increasing mRNA production and accumulating genome (vRNA) from nascent virions. The subsequent effect of this early virus multiplication was clearly visible in the kidney and heart, where LVI was consistently detected in greater quantities each day up until day 8 pi. The early LVI replication (as indicated by the specific detection of the replicative intermediate cRNA) and accumulation of nascent virus particles in the gills, prior to dissemination, probably accounts for this. Interestingly, the putatively non-virulent ISAV HPR0 is primarily associated with detection in the gills only [9,49]. The gill results suggest HVI progresses more slowly into a generalised infection. However, the fact that HVI may utilise an alternative entry point not examined in this study, cannot be excluded.

Virus virulence is a multifunctional trait and replication efficiency is one factor that has been associated with increased virulence. A study into the differential virulence mechanisms of two strains of IHNV of high or low virulence in salmonid fish was shown to be linked with faster in vivo kinetics and replication of the highly virulent virus [50,51]. Early innate host immune response was demonstrated to play a critical role and it was proposed that the lag in immune system stimulation might give the faster replicating highly virulent virus enough time to reach a threshold and outrun the host antiviral response [51]. In addition to the replication advantage, it was also established that the highly virulent IHNV had an advantage at the entry stage of the infection cycle, when data from immersion and IP-injection experiments were compared [52]. Contrary to this, investigations on simian immunodeficiency virus (SIV) have suggested high replication rates of the natural virus do not correspond with increased virulence [53]. In the present study following immersion challenge, whilst the LVI rapidly gained entry to gill cells and replicated immediately, it ultimately reached a lower peak viral load than the HVI and caused a lower mortality. We could argue this provides LVI with an evolutionary advantage over HVI. Replication to relatively high levels without killing all the available hosts may allow the virus to persist in a population and potentially spread further. However, further information on virus shedding rates is required to investigate this. Our data suggests that the LVI has an advantage at the entry stage in gills and also exhibited a higher replication rate during the early stages of infection. This finding agrees with previous experimental data demonstrating slower replication of a high pathogenic ISAV compared to a low pathogenic ISAV in Atlantic Salmon Kidney (ASK) cells at 20 °C [35]. The reason for apparent faster replication of LVI at early time points remains unknown, but could be due to enhanced viral RNA-dependant RNA polymerase (RdRp) activity. This activity could also be in conjunction with differential properties of the viral glycoproteins, such as more efficient particle attachment or membrane fusion, as demonstrated for highly pathogenic influenza A viruses [54,55]. At present, functional information on ISAV RdRp’s, and on the links between RdRp’s and surface proteins is limited.

The significant up-regulation of all four immune genes showed both LVI and HVI effectively stimulated the innate and adaptive immune systems. The response to both viruses was substantial and sustained. It was evident LVI disseminated more rapidly due to its replication at the earlier stages of infection, thus triggering an earlier systemic IFN-mediated immune response compared to that of HVI. Infection of TO cells with ISAV of low and high virulence suggested replication was a requirement for induction of immune genes [36]. The generation of dsRNA is a pivotal part of the replication process for many ssRNA viruses and longer stretches of dsRNA are key indicators of viral invasion to a cell [56]. In the present study, the detection of the replicative intermediary cRNA early in the gills signals its presence. Cells detect viral RNA and proteins via pathogen associated molecular pattern (PAMP) receptors, which in turn stimulate interferons and the antiviral response [32,57]. In this study, the more rapid systemic response induced by LVI might have provided a sufficient level of protection in a higher number of hosts, preventing this strain from reaching the damaging higher viral loads observed for HVI. In the latter half of the trial the immune genes of the HVI infected fish were up-regulated more in comparison to those of the LVI infected fish. Interferon responses to viral infection are usually transient and self-limited to avoid a prolonged anti-viral state which in itself can be detrimental to the host and interfere with haematopoiesis [57,58]. Indeed, the vast induction of cytokines and chemokines, generating a “cytokine storm” and overwhelming inflammatory responses, have been linked to highly pathogenic influenza virus pathogenesis [57,59,60]. The over-activation of IFNβ and tumour necrosis factor–α (TNFα) creates a powerful pro-inflammatory response compared to that of low pathogenic influenza viruses, tipping the balance of the response towards inflammation, contributing to tissue damage [61]. The possibility that the increased mortality caused by HVI, which coincided with high expression of immune markers was caused by similar immune mechanisms should not be excluded. This study only focussed on a very small aspect of the immune response, therefore a more in-depth analysis of a greater number of immune response genes in immersion challenged fish would be advantageous. What is clear from the present study, the immune response was sufficient to limit the infection by LVI while ineffectual at preventing HVI instigating a progressive infection causing an eventual fatal outcome.

RNA viruses have evolved diverse strategies to counteract and evade the host immune system. The influenza virus NS1 protein for example, is the primary antagonist of the innate immune response and remarkably effects many stages of the interferon response [62]. Two ISAV proteins, including the putative NS protein, have been linked to the antagonism of the IFN system [17,18], but it was not possible to relate these functions specifically here. In a study such as this, any viral IFN antagonistic effect within cells is likely to go unseen due to the vast cytokine inductions in neighbouring uninfected cells. Real-time PCR of segment 7 followed a similar profile to segment 8 in all three organs tested. However, correlation analysis suggested the segment 7 load, unlike segment 8, is unlikely to be directly related to the immune gene expression in either the gill or the heart. This has two possible explanations. Firstly, the functions of the proteins encoded by these segments are very different and the expression may be time dependent. In addition, the quantities of the required proteins are also substantially different and neither segment assay differentiates the two ORFs. The M protein is the most abundant protein in the virion [14], thus more is required compared to the non- or minor- structural proteins encoded by segment 7. Comparing the segment 7 to segment 8 ratios of both viruses indicated the potentially interesting observation that HVI appears to generate less segment 7 RNA than LVI. The significance of this is unknown and remains an area of further investigation, although may partly explain the lack of correlation between segment 7 and immune genes.

In conclusion, this study indicated that low virulent ISAV (LVI) replicated earlier in the gills and disseminated throughout the host more efficiently following immersion challenge compared to the highly virulent virus (HVI). This suggests potential variation in tissue or even cell tropism. Rapid replication in gills stimulated a marked systemic immune response that may have provided some protection against LVI-induced pathogenesis, causing a limited infection. In contrast, the host response against HVI was less effectual, allowing the virus to reach a higher load causing a progressive infection and ultimately inducing significantly more mortality. Further work is required to elucidate the underlying factors that allow LVI to replicate more efficiently in the gills following immersion infection. Other factors such as variable HE activity allowing increased viral particle liberation to infect more cells, or differing abilities in immune system interference may have a role to play. In addition, because the HVI does not appear to access the gills as quickly, other entry points such as skin, eye, gut, should also be investigated. A greater understanding of the molecular and pathophysiological mechanisms of virulence and pathogenesis of LVI and HVI is critical in developing strategies and preventative measures to combat viral infection by ISAV.

## Additional files

Additional file 1
**Primers and probes used in RT-qPCR and RNA specific RT-qPCR.** Table of details relating to all primer and probe sequences used in the standard real-time RT-PCR analysis and the RNA specific real-time RT-PCR analysis. Additional file 2
**Difference in expression of ISAV segment 7 compared to segment 8.** Expression ratio of segment 7 compared to segment 8 in gill (A), anterior kidney (B) and heart (C) in fish infected with either low virulent (LVI) or highly virulent (HVI) ISAV.
