# Supplementary material for: Low virulent infectious salmon anaemia virus (ISAV) replicates and initiates the immune response earlier than a highly virulent virus in Atlantic salmon gills
Source: Vet Res. 2014 Aug 21;45(1):83. doi: 10.1186/s13567-014-0083-x (PMC4144175; doi:10.1186/s13567-014-0083-x)
Supplement: Additional file 1 — Primers and probes used in RT-qPCR and RNA specific RT-qPCR. Table of details relating to all primer and probe sequences used in the standard real-time RT-PCR analysis and the RNA specific real-time RT-PCR analysis. [file 13567_2014_83_MOESM1_ESM.docx]

| **Name** | **Primer/probe** | **Sequence 5’-3’** | **Reference** |
| --- | --- | --- | --- |
|  | For | CCC CTC CAG GAC GTT TAC AAA |  |
| Elongation Factor 1α (ELF) | Rev | CAC ACG GCC CAC AGG TAC A | [21] |
|  | probe | ATC GGT GGT ATT GGA AC |  |
|  | For | CTA CAC AGC AGG ATG CAG ATG |  |
| ISAV segment 8 (seg8) | Rev | CAG GAT GCC GGA AGT CGA T | [21] |
|  | probe | CAT CGT CGC TGC AGT TC |  |
|  | For | CAG GGT TGT ATC CAT GGT TGA AAT G |  |
| ISAV segment 7 (seg7) | Rev | GTC CAG CCC TAA GCT CAA CTC | [21] |
|  | probe | CTC TCT CAT TGT GAT CCC |  |
|  | For | ACT GAA ACG CTA CTT CAA GAA GTT GA |  |
| Type I interferon, α1 & α2 (α/βIFN) | Rev | GCA GAT GAC GTT TTG TCT CTT TCC T | [33] |
|  | probe | CTG TGC ACT GTA GTT CAT TT |  |
|  | For | GAT GCT GCA CCT CAA GTC CTA TTA |  |
| Mx1 and Mx2 (Mx) | Rev | CGG ATC ACC ATG GGA ATC TGA | [33] |
|  | probe | CAG GAT ATC CAG TCA ACG TT |  |
|  | For | GCT GTT CAA CGG AAA ACC TGT TT |  |
| Type II interferon (γIFN) | Rev | GTC CAG AAC CAC ACT CAT CCA | [33] |
|  | probe | TCA CTG TCC TCA AAC GTG |  |
|  | For | CCA AAC ACT CCT GCC AGA ACA T |  |
| γIFN induced protein 10 (γIP) | Rev | ACA CTT CAT CCC TTT TCC GTT CTT | [33] |
|  | probe | CAG AGT GAC AAT GAT CTC C |  |
| s8-RT-mTAG | mRNA RT primer | CCA GAT CGT TCG AGT CGT TTT TTT TTT TTT TTT |  |
| s8-RT-cTAG | cRNA RT primer | GCT AGC TTC AGC TAG GCA TCA GTA AAA AAA GGC |  |
| mRNAtag-F | mRNA For | CCA GAT CGT TCG AGT CGT | [38] |
| cRNAtag-F | cRNA For | GCT AGC TTC AGC TAG GCA TC |  |
| s8-gle-m+cRNA-R | Rev (HVI) | TTG TTG TTT CAT TTC TCT GTG TTG AAT GG |  |
| s8-I141-m+cRNA-R | Rev (LVI) | TTG TTA ATT CAA TTC TAT GTG TTG TAT GG |  |
