# Supplementary figures and images for: Low virulent infectious salmon anaemia virus (ISAV) replicates and initiates the immune response earlier than a highly virulent virus in Atlantic salmon gills
Source: Vet Res. 2014 Aug 21;45(1):83. doi: 10.1186/s13567-014-0083-x (PMC4144175; doi:10.1186/s13567-014-0083-x)

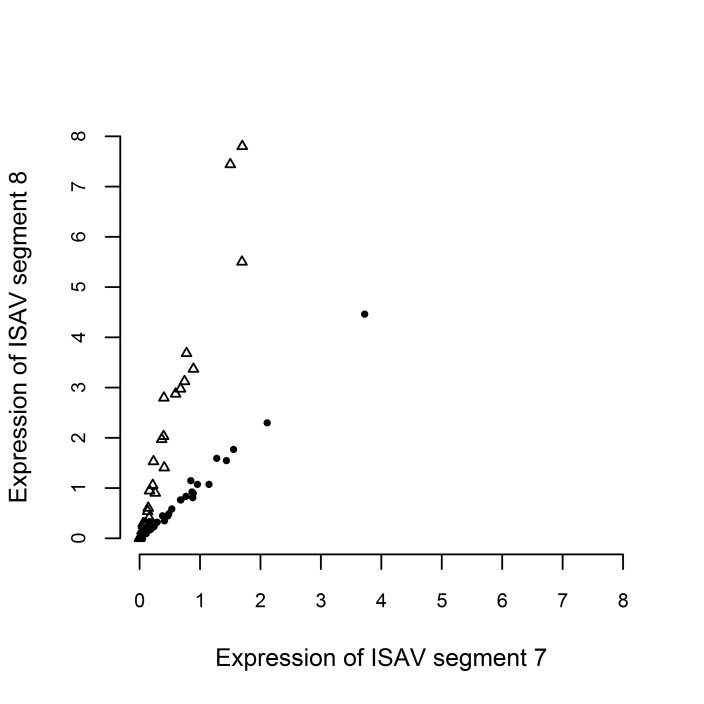

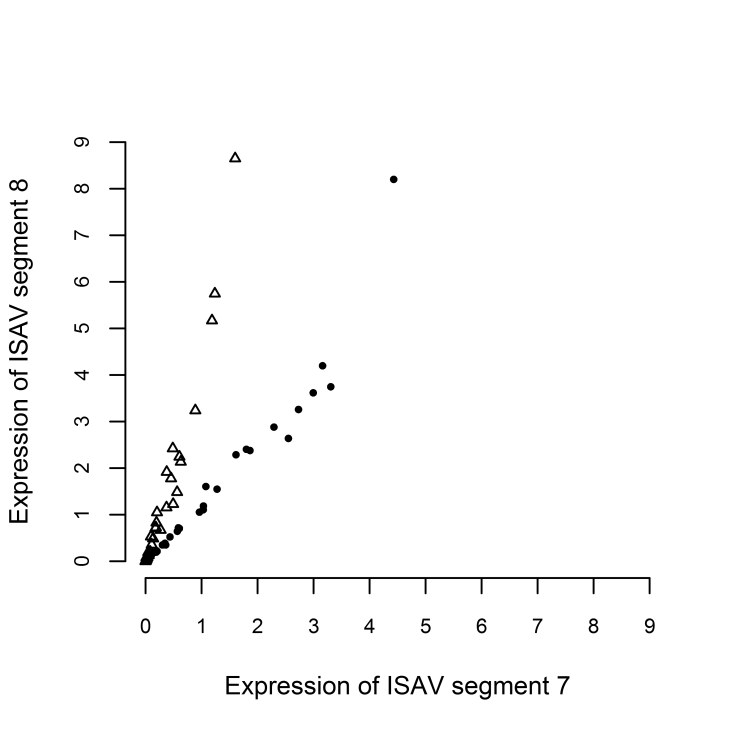


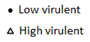


B)

A)

C)


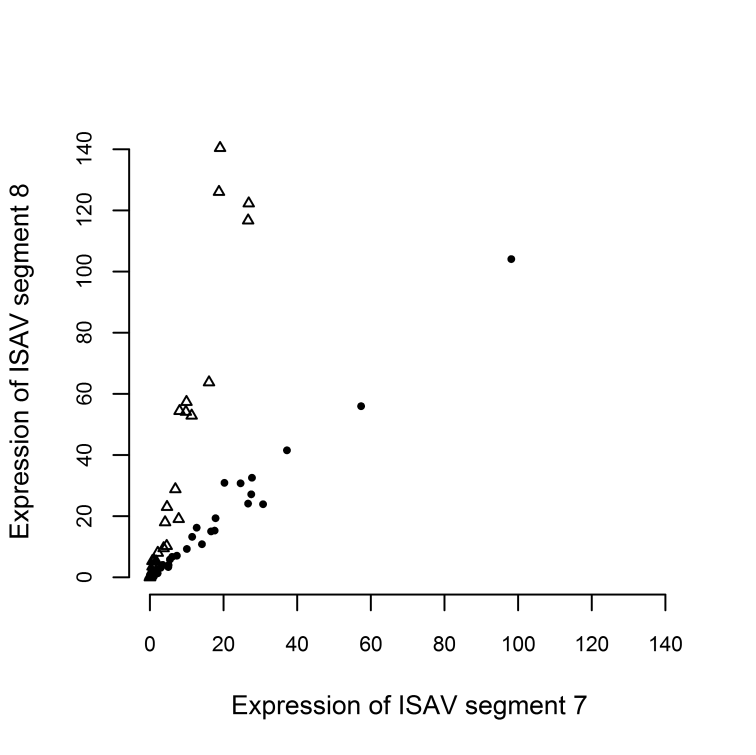

Supplement: Additional file 2 — Difference in expression of ISAV segment 7 compared to segment 8. Expression ratio of segment 7 compared to segment 8 in gill (A), anterior kidney (B) and heart (C) in fish infected with either low virulent (LVI) or highly virulent (HVI) ISAV. [file 13567_2014_83_MOESM2_ESM.docx]
